# Systematically *In Silico* Comparison of Unihormonal and Bihormonal Artificial Pancreas Systems

**DOI:** 10.1155/2013/712496

**Published:** 2013-10-24

**Authors:** Xiaoteng Gao, Huangjiang Ning, Youqing Wang

**Affiliations:** College of Information Science & Technology, Beijing University of Chemical Technology, Beijing 100029, China

## Abstract

Automated closed-loop control of blood glucose concentration is a daily challenge for type 1 diabetes mellitus, where insulin and glucagon are two critical hormones for glucose regulation. According to whether glucagon is included, all artificial pancreas (AP) systems can be divided into two types: unihormonal AP (infuse only insulin) and bihormonal AP (infuse both insulin and glucagon). Even though the bihormonal AP is widely considered a promising direction, related studies are very scarce due to this system's short research history. More importantly, there are few studies to compare these two kinds of AP systems fairly and systematically. In this paper, two switching rules, P-type and PD-type, were proposed to design the logic of orchestrates switching between insulin and glucagon subsystems, where the delivery rates of both insulin and glucagon were designed by using IMC-PID method. These proposed algorithms have been compared with an optimal unihormonal system on virtual type 1 diabetic subjects. The *in silico* results demonstrate that the proposed bihormonal AP systems have outstanding superiorities in reducing the risk of hypoglycemia, smoothing the glucose level, and robustness with respect to insulin/glucagon sensitivity variations, compared with the optimal unihormonal AP system.

## 1. Introduction

According to a prediction produced by the International Diabetes Federation (IDF) in 2009, there will be approximately 285 million diabetic subjects by 2010 and about 439 million diabetic subjects by 2030 [1, 2]. Diabetes has become a big issue that seriously harms human health. To regulate the blood glucose level [3, 4], exogenous insulin infusion is one of our main choices. In the recent decades, there has been a great deal of interest in developing a closed loop system that embodies an automated insulin delivery system [5] for regulating blood glucose (BG) in type 1 diabetes. Unlike intravenous (IV) administration, however, the subcutaneous (SC) insulin infusion presents a control challenge owing to a finite absorption rate of insulin analogs. This challenge may induce excessive drug accumulation in the subcutaneous tissue and then even hypoglycemia (blood glucose lower than 70 mg/dL).

Short-term hypoglycemia may cause more damage to patients' brain function and even cause death [6]. Therefore, minimizing or avoiding hypoglycemia is a daily challenge for diabetic patients. There are mainly three ways to rescue for hypoglycemia: mathematically minimizing the SC accumulation, suspending the insulin delivery (termed as prediction/suspending solution) [7, 8], and using glucagon as a counterregulatory agent in closed-loop system. The first method to handle hypoglycemia is to mathematically minimize the SC accumulation, for example, MPC [9–11]. We can design an objective function to optimally regulate the BG close to a target within the normoglycemic range and in the meantime to minimize the SC accumulation of insulin. Another strategy to avert hypoglycemia is a combination of glucose prediction [12] and insulin infusion suspending. The third solution is adding glucagon infusion [13].

Recently, El-Khatib et al. proposed a new scheme for glucose management [14]: dual subcutaneous infusion of insulin and glucagon, where a model prediction control (MPC) algorithm [15] was used to design the SC administration of insulin and a proportional-derivative control [16] was used to govern the glucagon infusion. Ward and his coworkers used the glucagon to prevent hypoglycemia in type 1 diabetes [17], where the fading memory proportional derivative (FMPD) algorithm was used to design the subcutaneous insulin and glucagon infusion rates. Several clinical trials [18, 19] have been finished and they demonstrate the feasibility and safety of BG control by a bihormonal artificial pancreas. However, in the literature [14, 17–20], the delivery rates for insulin and/or glucagon were determined separately. Obviously, separate design cannot exploit the full benefit of using bihormone and may result in hormone waste and even blood glucose fluctuation.

In this paper, a new method based on switching control theory [21, 22] and IMC-PID [23] controller was proposed to orchestrate switching between insulin and glucagon subsystems. Two kinds of switching rules were designed and compared in this paper [24]: P-type switching rule and PD-type switching rule. Compared with the reported bihormonal artificial pancreas systems [14, 17–20], the proposed algorithms have the following advantages. First, the proposed algorithms can coordinate the insulin and glucagon delivery simultaneously, such that more hypoglycemia can be minimized or avoided and more glucose concentrations can be kept within the normoglycemic range. Second, unreasonable operation, for example, delivering two hormones at the same time, was avoided in the new systems; hence, hormone waste and BG fluctuation can be minimized. Third, the transient phase (period during which neither insulin nor glucagon is delivered) can be further maximized, and the dosage of two hormones can be minimized. 

Even though there are a number of reported works on clinical testing of bihormonal AP system [14, 17–20], its superiority compared with the uni-hormonal AP system is not fully proved and widely accepted. For example, in a comment on the bihormonal therapy [25], it is stated that “in terms of glucose control, the results were quite similar to uni-hormonal systems.” It is further suggested in [25] that “the efficacy of glucagon should be tested compared to an insulin-only system”. Obviously, fair, systematic, and strict comparisons of the bihormonal and uni-hormonal AP systems are very important and valuable. However, strictly fair comparison of two therapies is impossible to carry out in clinic due to inevitable disturbances. Because any disturbance can be avoided in computer simulation, *in silico* test provides a possibility to fairly, systematically, and strictly compare of the bihormonal and uni-hormonal AP systems. In the *in silico* trials, various therapies can be tested in the same scenario.

Because IMC-PID is used to design the insulin and glucagon delivery rates in the proposed bihormonal AP systems, IMC-PID is also implemented in the uni-hormonal benchmark AP system. As introduced in the previous paragraph, adding prediction/suspending term can enhance the performance of the uni-hormonal AP system. There have been a number of reported studies for hypoglycemia prediction [7, 8]. To achieve the performance limitation of the uni-hormonal AP system with prediction/suspending term, the perfect prediction/suspending solution is included in the uni-hormonal benchmark AP system; hence, this system can be considered as an optimal uni-hormonal AP system. To the authors' best knowledge, this is the first work to compare the bihormonal AP system and the optimal uni-hormonal AP system fairly and systematically.

The rest of this paper is organized as follows. In Section 2, the simulation models were briefly introduced. In Section 3, the IMC-PID controller was described and two switching rules were also proposed for blood glucose management. As a comparative standard, the optimal prediction/suspending therapy was presented for minimizing hypoglycemia episodes. In Section 4, the simulation results demonstrated the feasibility and safety of BG regulation using the switching control theory; statistics results can be used to further compare these therapies quantitatively; the robustness of these therapies was compared with respect to meals size variations, insulin/glucagon sensitivity variations, and measurement noises. In Section 5, to test its robustness with respect to intersubject variability, the proposed therapies were tested on ten virtual subjects. Finally, Section 6 concludes this note.

## 2. Brief Introduction on Simulation Models

The dynamic model for a virtual subject [26] consists of a glucose subsystem, an insulin subsystem, a meal subsystem, and a glucagon subsystem. The detailed descriptions for the glucose and insulin subsystems were introduced in [27, 28], respectively; the meal subsystem was presented in the paper [29]. The glucagon subsystem was introduced in the paper [30].

**Figure 1:**
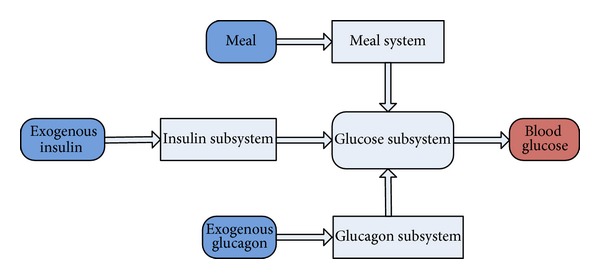
The block diagram of simulation models. The simulation models for a virtual subject consist of a glucose subsystem, an insulin subsystem, a meal subsystem, and a glucagon subsystem.

The block diagram of the virtual subject is shown in Figure 1.

### 2.1. Glucose Subsystem

The glucose subsystem is described by the following four equations [27]:

(1) G(t)=Q1(t)VG,dQ1(t)dt=−[F01cVGG(t)+x1(t)]Q1(t)+k12Q2(t)−FR+UG(t)+EGP0[1−x3(t)],dQ2(t)dt=x1(t)Q1(t)+[k12+x2(t)]Q2(t),F01c={F01G(t)≥81 mg/dL,F01G(t)4.5otherwise,FR={0.003(G−9)VGG(t)≥162 mg/dL,0otherwise,UG(t)=DGAGte−t/tmax⁡,Gtmax⁡,G2,

where *V*_*G*_ is the distribution volume of the accessible compartment and *Q*_1_ and *Q*_2_ are the masses of glucose in the accessible and nonaccessible compartments, respectively. *G*(*t*) represents the glucose (measurable) concentration (mg/dL), *k*_12_ represents the transfer rate constant between *Q*_1_ and *Q*_2_, EGP_0_ is endogenous glucose production (EGP) extrapolated to the zero insulin concentration, *F*_01_^*c*^ is the amount of noninsulin-dependent glucose corrected for the ambient glucose concentration, and *F*_*R*_ is the rate of renal glucose clearance above the glucose threshold of 162 (mg/dL). *U*_*G*_ represents the gut absorption rate, *t*_max⁡,*G*_ is the time of maximum appearance rate of glucose in the accessible glucose compartment, and *D*_*G*_ and *A*_*G*_ represent the amount of carbohydrates digested and carbohydrate bioavailability, respectively.

### 2.2. Insulin Subsystem

The insulin subsystem is described as follows [28]:

(2.2) $$\begin{array}{c} \frac{dQ_{1a}}{dt}=ku-k_{a1}Q_{1a}-\text{L}{\text{D}}_{a},\\ \frac{dQ_{1b}}{dt}=\left(1-k\right)u-k_{a2}Q_{1b}-\text{L}{\text{D}}_{b},\\ \frac{dQ_{2}}{dt}=k_{a1}*\left(Q_{1a}-Q_{2}\right),\\ \frac{dQ_{3}}{dt}=k_{a1}Q_{2}+k_{a2}Q_{1b}-k_{e}Q_{3},\\ \text{L}{\text{D}}_{a}=\frac{V_{\text{MAX},\text{LD}}Q_{1a}}{\left(k_{M,\text{LD}}+Q_{1a}\right)},\\ \text{L}{\text{D}}_{b}=\frac{V_{\text{MAX},\text{LD}}Q_{1b}}{\left(k_{M,\text{LD}}+Q_{1a}\right)}, \end{array}$$

where *Q*_1*a*_ represents mass of insulin administered as continuous infusion, *Q*_1*b*_ is the mass of insulin given as a bolus, *k* represents the proportion of the total input flux passing through the slower, two compartment channel, *k*_*a*1_ and *k*_*a*2_ represent transfer rates, LD_*a*_ and LD_*b*_ are local degradation at the injection site for continuous infusion and bolus, *Q*_2_ and *Q*_3_ are the dose of in the nonaccessible subcutaneous compartments and the plasma compartment, respectively. *k*_*M*,LD_ represents the amount of insulin mass in which insulin degradation is equal to half of its maximal value for continuous infusion and bolus, and *V*_MAX,LD_ represents the saturation level describing Michaelis-Menten dynamics of insulin degradation for continuous infusion and bolus.

### 2.3. Meal Subsystem

The meal subsystem is described by [29, 31, 32]. This model describes glucose transit through the upper small intestine and stomach. Consider

(3) $$\begin{array}{c} {\dot{q}}_{\text{sto}1}\left(t\right)=-k_{21}*q_{\text{sto}1}\left(t\right)+D\delta \left(t\right),\\ {\dot{q}}_{\text{sto}2}\left(t\right)=-k_{\text{empt}}*q_{\text{sto}2}\left(t\right)+k_{21}*q_{\text{sto}1}\left(t\right),\\ {\dot{q}}_{\text{gut}}\left(t\right)=-k_{\text{abs}}*q_{\text{gut}}\left(t\right)+k_{\text{empt}}*q_{\text{sto}2}\left(t\right),\\ q_{\text{sto}}\left(t\right)=q_{\text{sto}1}\left(t\right)+q_{\text{sto}2}\left(t\right), \end{array}$$

where *q*_sto_ and *q*_gut_ present the amounts of glucose in the stomach and intestine, respectively; *q*_sto1_ and *q*_sto2_ are the solid and liquid phase, respectively; *δ*(*t*) presents the impulse function; *D* presents the amount of ingested glucose; *k*_21_ and *k*_empt_ are the rates of grinding and gastric emptying, respectively; the *k*_abs_ present the rate constant of intestinal absorption.

### 2.4. Glucagon Subsystem

At the heart of the model is a two-compartment representation of glucagon kinetics [30]:

(4) $$\begin{array}{c} {\dot{h}}_{1}=\frac{\left(u*k_{g1}*\text{AG}-h_{1}\right)}{\left(k_{g2}*t_{\max,G}\right)},\\ {\dot{h}}_{2}=\frac{\left(h_{1}-h_{2}\right)}{\left(k_{g2}*t_{\max,G}\right)},\\ h=h_{1}+h_{2}, \end{array}$$

where *h* is the glycogen conversion rate; *t*_max⁡,*G*_ and AG present the time of maximum appearance rate of glucose and glucagon bioavailability, respectively; *k*_*g*1_ and *k*_*g*2_ are transfer constants which affect the transfer speed. In Section 4, *k*_*g*1_ and *k*_*g*2_ are chosen as 1.50 and 0.63 for standard subject, respectively.

## 3. Controller Design

### 3.1. IMC-PID Controller

Due to its simple structure and excellent robustness, PID controller finds widespread implementation in the industrial process control. Therefore, a great deal of effort has been directed at designing the best turning parameters for different process models. Among various PID-tuning methods, internal model control (IMC) theory has greatly succeeded because of its simplicity, good robustness, and successful practical applications [33]. For short, this kind of PID is named IMC-PID. It is well known that different patients have various dynamics, so finding suitable turning parameters is a great challenge for clinical application. One promising candidate to handle this challenge is IMC-PID, because it has only one adjustable parameter *λ*. 

**Figure 2:**
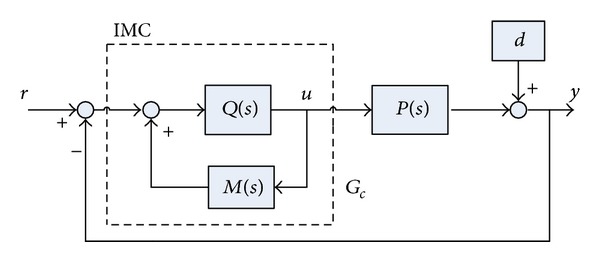
The structure of IMC controller, where the part inside the dashed frame is a PID controller [33].

The structure of the IMC controller is demonstrated in Figure 2, where *r*, *y*, and *d* are the input, output, and load disturbances, respectively. *P*(*s*) is the controlled subject, *M*(*s*) is the subject's model, and *Q*(*s*) = *M*_−_^−1^(*s*)*F*(*s*) (*M*_−_(*s*) is the minimum phase portion of *M*(*s*) and *F*(*s*) is a designed filter). In Figure 2, the part inside the dashed frame is a PID controller, *Q*_*c*_, described by the following equation:

(5) $$\begin{array}{c} Q_{c}=\frac{M_{-}^{-1}\left(s\right)F\left(s\right)}{1-M_{-}^{-1}\left(s\right)F\left(s\right)M\left(s\right)},\\ F\left(s\right)=\frac{1}{{\left(\lambda s+1\right)}^{n}}. \end{array}$$

From (5), one can determine the parameters of the PID controller. The order *n* is decided by the order of the minimum phase portion (*M*_(*s*)). The tradeoff between robust stability and output tracking performance can be balanced by the parameter *λ*. For smaller value of *λ*, the closed-loop system will has better output tracking performance but worse robustness, vice versa. The simulation results in Section 4 demonstrate that the proposed algorithm has great robustness.

### 3.2. Perfect Predictive Algorithm and Insulin Pump Suspension

According to the published studies, we have observed that the risk of “overcorrection hypoglycemia” is the greatest after a large amount of insulin. The simplest strategy to reduce hypoglycemia is to interrupt insulin delivery. It is clear that a key factor for suspending insulin infusion is having an accurate glucose predictor. Because virtual subjects were used in this study, the future glucose level can be exactly predicted using the subjects' accurate model. Hence, the optimal time for the insulin infusion suspension can be obtained such that the lowest BG value is exactly higher than 70 mg/dL. 

The dosing of insulin *u* was determined by the following equation:

(6) $$\begin{array}{c} u=u_{0}+u_{\text{PID}}, \end{array}$$

where *u*_0_ is the basal insulin delivery rate [35, 36] and *u*_PID_ is designed by using IMC-PID controller.

If a pending hypoglycemia event was alarmed by the perfect predictive algorithm, the insulin delivery rate *u* will be forced to zero. Obviously, the above-mentioned algorithm can be considered the optimal uni-hormonal system. 

### 3.3. Switched Control System

**Figure 3:**
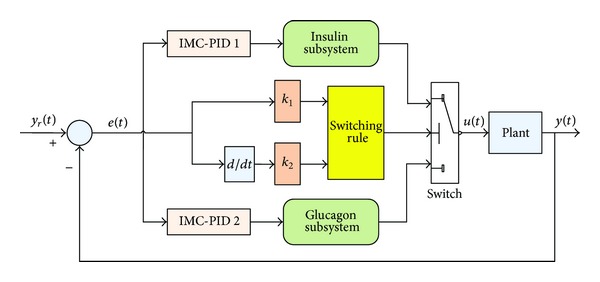
The structure of the proposed switched system.

The switched control system in this study is a dynamic system that consists of two subsystems and a switching law [37] that orchestrates switching between these two subsystems [38]. The structure of the switched controller is shown in Figure 3.

Two kinds of switching rules [39] are proposed in this study as shown in the following equations.

(1) P-type switching rule: consider

(7) $$\begin{array}{c} \delta \left(t\right)=\{\begin{array}{cc} 1 & \text{if}\;e\geq k_{e1}\;\text{mg}/\text{dL},\\ 0 & \text{if}\;e\leq -k_{e2}\;\text{mg}/\text{dL}, \end{array} \end{array}$$

where *δ*(*t*) = 1 indicates insulin subsystem active mode and *δ*(*t*) = 0 indicates glucagon subsystem active mode; *e* is the blood glucose tracking error; the positive constants *k*_*e*1_ and *k*_*e*2_ are thresholds for the switching controller.

(2) PD-type switching rule: condider

(8) $$\begin{array}{c} \delta \left(t\right)=\{\begin{array}{cc} 1 & \text{if}\;ds\geq k_{s1},\\ 0 & \text{if}\;ds\leq -k_{s2}, \end{array}\\ ds=e+k_{s}\frac{de}{dt}, \end{array}$$

where *k*_*s*1_ and *k*_*s*2_ are thresholds; *k*_*s*_ > 0 is a designed parameter. Substantially, *ds* can be considered a prediction of the future tracking error *e*, where *k*_*s*_ is the prediction horizon.

## 4. *In Silico* Tests on Standard Virtual Subject

Based on metabolism model introduced in Section 2 and its suggested parameter settings in the literature [27–30], one virtual subject was built. For convenience, this subject was named standard subject. All simulation tests in Section 4 were finished on the standard subject. The test duration for each algorithm is 24 hours. Four kinds of closed-loop control methods were tested in each study: insulin-only therapy, prediction/suspending therapy, P-type dual infusion therapy, and PD-type dual infusion therapy. Total carbohydrate consumption consists of breakfast, lunch, and dinner (40, 60, and 85 g of carbohydrates, taking at 7:00, 12:00, and 18:00, resp.).

### 4.1. Nominal Case

**Figure 4:**
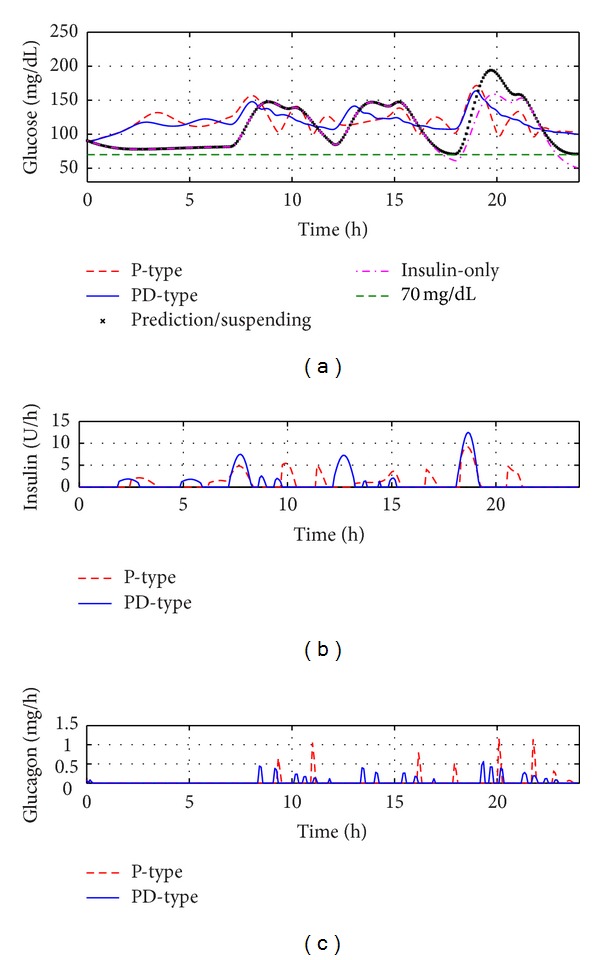
Glucose management results of the standard subject under four algorithms, where the whole testing duration is 24 hours. (a) It shows all BG curves. (b) It shows the corresponding insulin infusion rate determined by switching controllers (P-type dual infusion therapy and PD-type dual infusion therapy, resp.). (c) It shows the corresponding glucagon infusion rate determined by switching controllers (P-type dual infusion therapy and PD-type dual infusion therapy, resp.).

All simulation tests consist of four cases. There is only insulin infusion determined by *u* = 0.42 + *u*_PID_ in the first case, where *u*_PID_ is designed by using IMC-PID controller.  Figure 4(a) shows the closed-loop control performance of the insulin-only therapy, where *λ* = 0.40 was used for the tuning parameter. However, the control result is still not good enough. Hypoglycemia occurred during the closed-loop test. 

The second case is testing the glucose prediction and insulin suspension therapy. Through the combination of basal insulin and accurate suspending, tighter control of blood glucose can be achieved compared with the insulin-only therapy. As shown in Figure 4(a), the prediction/suspending therapy has superior capability to avoid hypoglycemia compared with the insulin-only therapy. The control algorithm can be described by

(9) $$\begin{array}{c} u=\{\begin{array}{cc} 0.42+u_{\text{PID}}, & \text{no}\;\text{hypoglycemia}\;\text{alarm},\\ 0, & \text{have}\;\text{hypoglycemia}\;\text{alarm}. \end{array} \end{array}$$

The P-type switching controller was used in the third experiment. The dosing of insulin and glucagon was determined by IMC-PID controllers, respectively. The switching rule was demonstrated by the following equations:

(10) $$\begin{array}{c} \delta \left(t\right)=\{\begin{array}{cc} 1 & \text{if}\;e>5\;\text{mg}/\text{dL},\\ 0 & \text{if}\;e<-5\;\text{mg}/\text{dL}. \end{array} \end{array}$$

The fourth strategy is PD-type switching therapy. The dosing of insulin and glucagon was designed by IMC-PID controllers, respectively. The switching rule was described by

(11) $$\begin{array}{c} \delta \left(t\right)=\{\begin{array}{cc} 1 & \text{if}\;ds>15,\\ 0 & \text{if}\;ds<-15. \end{array} \end{array}$$

The excellent glycemic control results under two switching controllers (P-type and PD-type) were presented in Figure 4(a) while the designed insulin and glucagon delivery rates were shown in Figures 4(b) and 4(c), respectively.

Some statistical results in Table 1 can be used to further compare the above-mentioned therapies quantitatively. The following results demonstrate that the switched therapy based on PD-type switching rule has the best glycemic control performance among these therapies. 

### 4.2. Robustness Analysis

The robustness of a BG management therapy is a significant factor for clinical application. Some uncertainties of external conditions may lead to severe BG fluctuation. For example, the meal size and the sensitivities of insulin, and glucagon may change dramatically.

**Figure 5:**
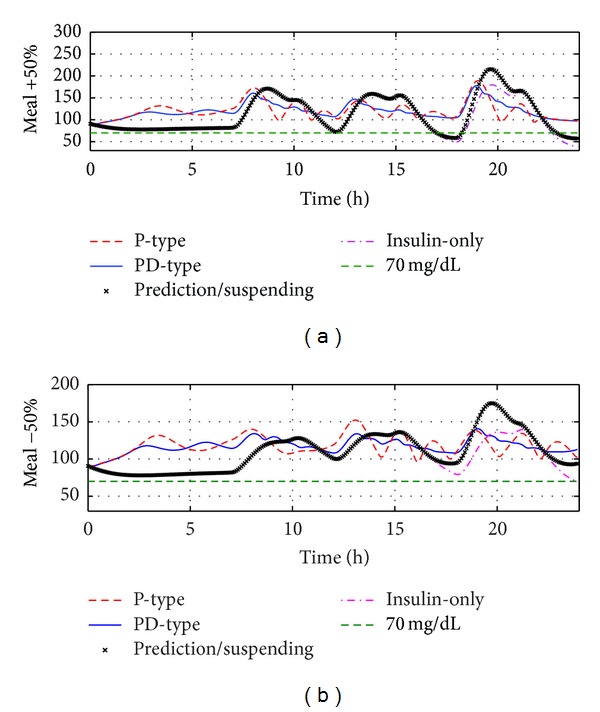
Comparison of four algorithms' robustness with respect to meal size variations on the standard subject. (a) There are +50% variations on meal sizes; (b) there are −50% variations on meal sizes.

**Figure 6:**
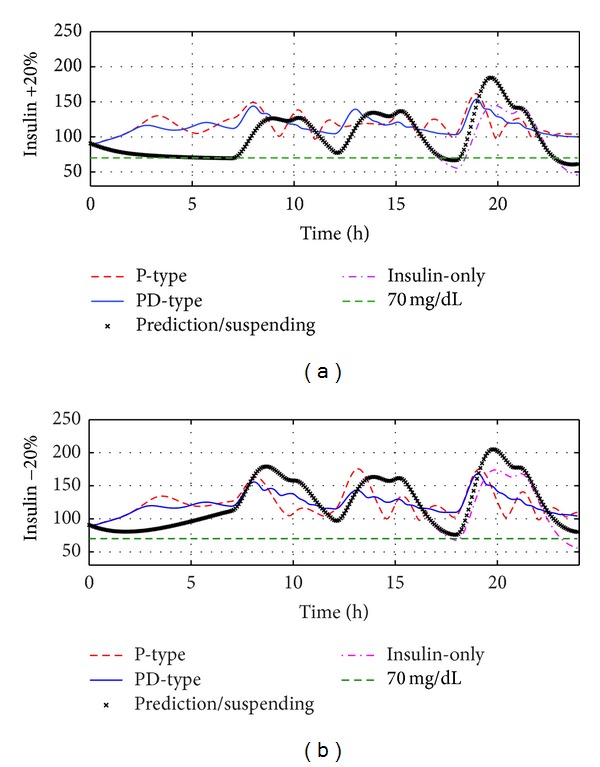
Comparison of four algorithms' robustness with respect to insulin sensitivity variations on the standard subject. (a) There are +20% variations on insulin sensitivity; (b) there are −20% variations on insulin sensitivity.

**Figure 7:**
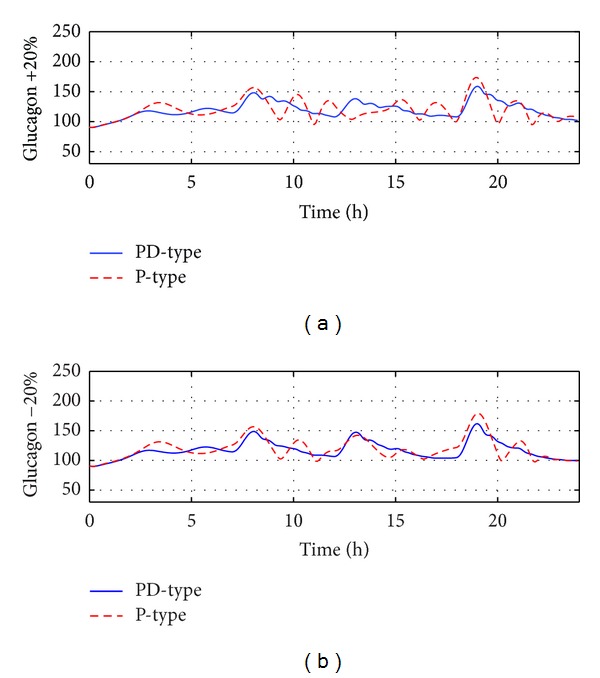
Comparison of two algorithms' robustness with respect to glucagon sensitivity variations on the standard subject. (a) There are +20% variations on glucagon sensitivity; (b) there are −20% variations on glucagon sensitivity.

In the sequel, the robustness analysis consists of four components: studies on meal size variations, insulin sensitivity variations, glucagon sensitivity variations, and measurement noises. Figures 5, 6, and 7 show the BG curves under four therapies with meal size uncertainties, insulin sensitivity uncertainties, and glucagon sensitivity uncertainties, respectively, where the PD-type switching therapy has the best closed-loop control performance. The following results can help people to evaluate the robustness and safety of the proposed switching therapies. Some detailed statistical results were given in Tables 2, 3, and 4. When there are variations on meal sizes or insulin sensitivity, both insulin-only and prediction/suspending therapies are faced with increased hypoglycemia and hyperglycemia events; however, both P-type and PD-type therapies can keep all glucose concentrations within the safe range (70~180 mg/dL). This demonstrates that the bihormonal AP system has superior robustness with respect to meal sizes and insulin sensitivity variations compared with the uni-hormonal AP system. In all six situations, PD-type therapy always has smaller BGI and SD values compared with P-type therapy. That is to say, PD-type therapy can control the glucose level more smoothly.

**Figure 8:**
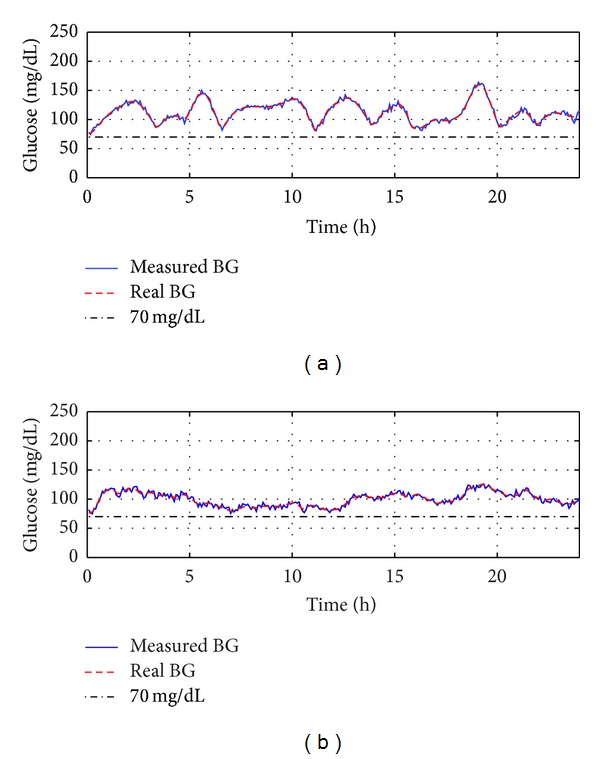
Closed-loop control results with measure noises: (a) Blood glucose curves under the P-type therapy; (b) blood glucose curves under the PD-type therapy.

In clinical practice, the inaccuracy of the continuous glucose monitoring system (CGMS) enormously affects the performance of the closed-loop control system. To simulate the measurement noises, white Gaussian noises with zero mean and $\sqrt{3}$ standard deviation have been added on the output. The closed-loop control results with measurement noises are shown in Figure 8. One can see that all blood glucose values can be kept in the safe range even though there are measurement noises, which demonstrates that the proposed method has good robustness with respect to measurement noises. In all five situations, PD-type therapy always has smaller BGI and SD values compared with P-type therapy. That is to say, PD-type therapy can control the glucose level more smoothly. 

## 5. *In Silico* Tests on Group Subjects

### 5.1. Method to Create Group Virtual Subjects

In order to test whether the controller performed well for different subjects, the proposed strategy is evaluated on ten virtual subjects. Model parameters represent the high intersubject and temporal variation in insulin needs in type 1 diabetic subjects. Different combinations of free parameters in the metabolism model represent different virtual patients. These parameters are assumed to be log-normally distributed to ensure their non-negativity. Ten virtual subjects were generated using the joint distribution; that is, ten realizations of the log-transformed parameter vector were randomly extracted from the multivariate normal distribution characterizing intersubject variability. Finally, the parameters in these ten virtual subjects were obtained by using antitransformation [40]. In our opinion, these ten subjects could represent a wide range of type 1 diabetic population.

### 5.2. Simulation Results

**Figure 9:**
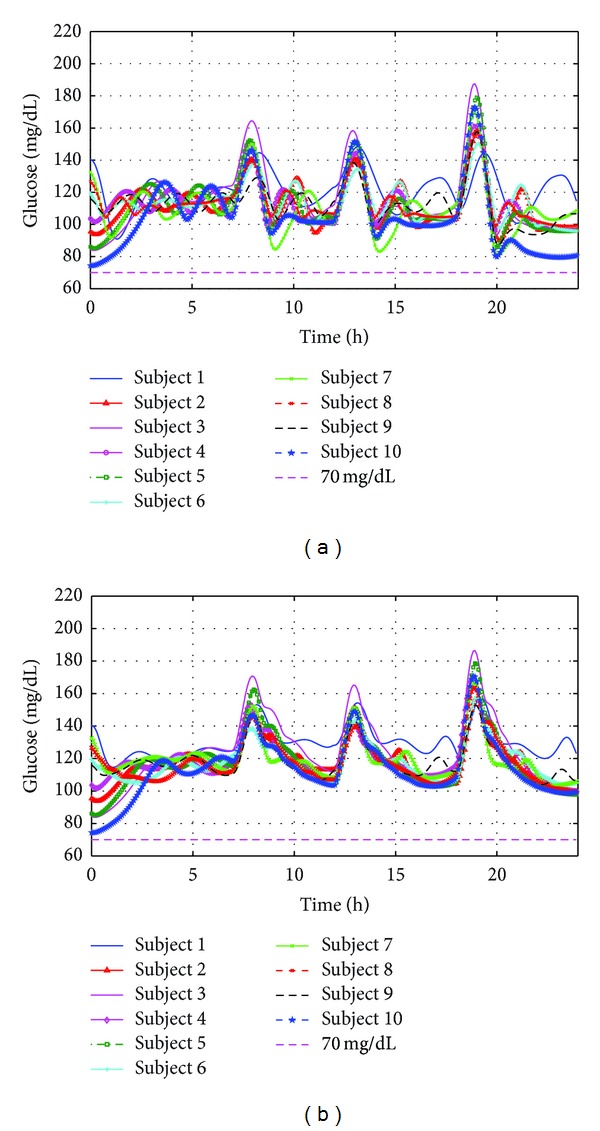
The blood glucose curves of ten virtual subjects under two proposed therapies: (a) P-type therapy; (b) PD-type therapy.

**Figure 10:**
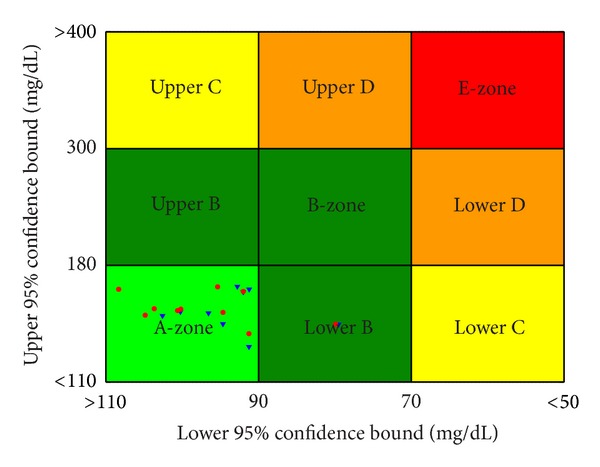
The control-variability grid analysis (CVGA) plot for these two proposed therapies: 90% in A-zone and 10% in B-zone for P-type therapy (*▼*); 90% in A-zone and 10% in B-zone for PD-type therapy (■).

All simulation results were demonstrated in BG response curves and control-variability grid analysis (CVGA) plots [41]. All of these ten subjects followed a two-day scenario. The BG response curves and CVGA plot in the second day (under the proposed therapies) are given in Figures 9 and 10, respectively. From Figure 9, one can see that all subjects have no hypoglycemic event and only one tiny hyperglycemic event under two therapies. In addition, 90% of subjects are within A-zone under both P-type and PD-type therapies as shown in Figure 10. These results indicate that these proposed closed-loop control algorithms can provide satisfactory BG control, and hence it is an excellent candidate for tight blood glucose control.

## 6. Conclusions

Two novel methods based on switched control theory, P-type and PD-type switching therapies, were proposed for the bihormonal AP system. Systematic *in silico* tests demonstrate that these proposed bihormonal AP systems have superior glycemic control performance than the optimal uni-hormonal AP system. Using the bihormonal AP system, all glucose concentrations can be kept within the safe range. In addition, these proposed switching therapies have excellent robustness with respect to meal size variations, insulin/glucagon sensitivity variations, measurement noises, and intersubject variability. In terms of BGI and SD, the PD-type therapy is better than the P-type one. Hence, the bihormonal AP system based on switching control theory is a promising direction for BG management. 

## Figures and Tables

**Table 1:** Statistical comparison for the insulin-only, prediction/suspending, P-type, and PD-type therapies on the standard subject, where BGI is the blood glucose index [34] and SD is the standard deviation of corresponding glucose curve.

Therapies	% of BG <70 mg/dL	% of BG >180 mg/dL	BGI	SD (mg/dL)
Insulin-only therapy	8	0	3.43	30.93
Prediction/suspending	0	4	3.13	33.60
P-type therapy	0	0	0.70	16.28
PD-type therapy	0	0	0.55	14.24

**Table 2:** Statistical results for experiments on the standard subject under the insulin-only, prediction/suspending, P-type, and PD-type therapies, when there are +50% or −50% variations on meal sizes.

Meal size	Therapies	% of BG <70 mg/dL	% of BG >180 mg/dL	BGI	SD (mg/dL)
+50%	Insulin-only therapy	11	0	5.29	38.84
	Prediction/suspending	9	6	4.64	41.39
	P-type therapy	0	0	1.00	20.64
	PD-type therapy	0	0	0.81	17.73

−50%	Insulin-only therapy	0	0	1.95	21.98
	Prediction/suspending	0	0	1.99	25.97
	P-type therapy	0	0	0.51	13.18
	PD-type therapy	0	0	0.30	9.74

**Table 3:** Statistical results for experiments on the standard subject under the insulin-only, prediction/suspending, P-type, and PD-type therapies. There are two situations: +20% means that the insulin sensitivity is 20% higher than the nominal case; −20% means that the insulin sensitivity is 20% lower than the nominal case.

Insulin sensitivity	Therapies	% of BG <70 mg/dL	% of BG >180 mg/dL	BGI	SD (mg/dL)
+20%	Insulin-only therapy	16	0	4.35	27.89
	Prediction/suspending	13	2	3.97	31.23
	P-type therapy	0	0	0.54	14.38
	PD-type therapy	0	0	0.41	12.67

−20%	Insulin-only therapy	6	0	3.31	35.64
	Prediction/suspending	0	6	3.19	37.25
	P-type therapy	0	0	1.08	20.26
	PD-type therapy	0	0	0.79	15.51

**Table 4:** Statistical results for experiments on the standard subject under P-type and PD-type therapies. There are two situations: +20% means that the glucagon sensitivity is 20% higher than the nominal case; −20% means that the glucagon sensitivity is 20% lower than the nominal case.

Glucagon sensitivity	Therapies	% of BG <70 mg/dL	% of BG >180 mg/dL	BGI	SD (mg/dL)
+20%	P-type therapy	0	0	0.74	16.73
	PD-type therapy	0	0	0.56	13.72

−20%	P-type therapy	0	0	0.79	17.90
	PD-type therapy	0	0	0.56	14.71
